# Histone deacetylase inhibitor MPT0B291 suppresses Glioma Growth *in vitro* and* in vivo* partially through acetylation of p53

**DOI:** 10.7150/ijbs.45505

**Published:** 2020-10-19

**Authors:** Batsaikhan Buyandelger, Eli E Bar, Kuo-Sheng Hung, Ruei-Ming Chen, Yung-Hsiao Chiang, Jing-Ping Liou, Huei-Mei Huang, Jia-Yi Wang

**Affiliations:** 1 Graduate Institute of Medical Sciences, College of Medicine, Taipei Medical University, 110 Taipei, Taiwan.; 2 Department of Neurology, Mongolian National University of Medical Sciences, 14210 Ulaanbaatar, Mongolia.; 3 Department of Pathology and Neurosurgery, University of Maryland School of Medicine, 21201 Baltimore, MD, USA.; 4 Department of Neurosurgery, Wan Fang Hospital, Taipei Medical University, 116 Taipei, Taiwan.; 5 Department of Neurosurgery, Taipei Medical University Hospital, Taipei Medical University, 110 Taipei, Taiwan.; 6 Neuroscience Research Center, Taipei Medical University, 110 Taipei, Taiwan.; 7 School of Pharmacy, College of Pharmacy, Taipei Medical University, 110 Taipei, Taiwan.

**Keywords:** glioma, HDAC6 inhibition, cell death, cell cycle arrest, xenograft, allograft

## Abstract

**Background:** Histone deacetylase (HDAC) inhibitors have emerged as a new class of anti-tumor agents for various types of tumors, including glioblastoma.

**Methods and results:** We found that a novel HDAC inhibitor, MPT0B291, significantly reduced the cell viability and increased cell death of human and rat glioma cell lines, but not in normal astrocytes. We also demonstrated that MPT0B291 suppressed proliferation by inducing G1 phase cell cycle arrest and increased apoptosis in human and rat glioma cell lines by flow cytometry and immunocytochemistry. We further investigated the anti-tumor effects of MPT0B291 in xenograft (mouse) and allograft (rat) models. The IVIS200 images and histological analysis indicated MPT0B291 (25 mg/kg, *p. o.*) reduced tumor volume. Mechanistically, MPT0B291 increased phosphorylation and acetylation/activation of p53 and increased mRNA levels of the apoptosis related genes PUMA, Bax, and Apaf1 as well as increased protein level of PUMA, Apaf1 in C6 cell line. The expression of cell cycle related gene p21 was also increased and Cdk2, Cdk4 were decreased by MPT0B291.

**Conclusion:** Our study highlights the anti-tumor efficacy of a novel compound MPT0B291 on glioma growth.

## Introduction

Glioblastoma multiforme (GBM), classified as a grade IV astrocytoma by the World Health Organization, is the most common and aggressive primary brain tumor in adults 1. GBM has a poor prognosis, and the median survival time in patients with GBM is approximately 12-15 months 2. Temozolomide (TMZ) is the first-line drug for the treatment of malignant gliomas, but it results in only a small increase in the overall survival time in patients with glioma. TMZ resistance has become a major problem, therefore, new therapeutic approaches for GBM are urgently needed 3.

Histone Deacetylase (HDAC) inhibitors are multi-targeted therapeutic compounds 4, 5. HDACs exert epigenetic control on transcriptional activity by removing negatively charged acetyl groups from lysine residues in histones, which causes chromatin condensation and limits the accessibility of transcription factors to DNA. HDACs can also deacetylate non-histone proteins 6 such as the transcription factor p53. Interactions of p53 and HDACs result in p53 deacetylation, thereby reducing its transcriptional activity 7. Accordingly, HDAC inhibitors cause increased p53 acetylation and p53 dependent activation of apoptosis and senescence.

HDAC inhibitors are potential anti-cancer drugs because of their ability to induce cell cycle arrest, cell differentiation, and apoptosis as well as to attenuate metastasis in various cancer cell types 8. Suberoyl anilidehydroxamic acid (SAHA), an HDAC inhibitor, suppresses the growth of gliomas and is currently being evaluated in a phase III clinical trial 9. Among the 18 mammalian HDACs, HDAC6 is unique for being localized exclusively in the cytoplasm and possesses two tandem catalytic deacetylase domains 10. Furthermore, HDAC6 is crucial as a target for neuroprotection and neuroregeneration in traumatic brain injury, stroke, and neurodegenerative disorders 11-14. We are therefore interested in drug development targeting HDAC6. We previously found that azaindolylsulfonamide (now named MPT0B291) selectively inhibits HDAC6 activity in colorectal cancer cells. We previously developed a series of indolyl/azaindolylsulfonylcinnamic hydroxamate (compound 7-15) and evaluated for bioactivity *in vitro* and *in vivo*. A SAR analysis revealed that compound 12, a 7-azaindole, renamed as MPT0B291, exhibited activity superior to that of indole, indazole, 6-azaindole, and 7-azaindoline. Compound 12 also possessed anti-proliferative activity with IC50 values of 0.39-2.51 μM against 11 diverse human cancer cell lines. MPT0B291 desplayed 60-fold selectivity for HDAC6 over HDAC1 and 223-fold over HDAC2, which is 5 and 22 times more selective than ACY1215. MPT0B291 showed greater antitumor activity (55.8% and 66.9% TGI in 25 and 100 mg/kg, *p.o.*) than SAHA (with 48.4% TGI in 200 mg/kg, *p.o.*) 15.

In this study, we demonstrated that a novel HDAC inhibitor, MPT0B291 induced cell death and cell cycle arrest as well as suppressed cell proliferation in C6 and U-87MG cell lines (*in vitro*) and in xenograft as well as allograft animal models (*in vivo*). The transcription factor p53 controls the expression of genes affecting those cellular processes such as proliferation, DNA repair, programmed cell death (apoptosis), and cell migration 16 and is important in glioma biology 17. GoPubMed text mining analysis suggested that the tumor suppressor gene p53 is associated with these processes and may be affected by MPT0B291. The p53 induces senescence, apoptosis, and DNA repair by controlling its target genes including p21, Bax, PUMA and more 16. Furthermore, we hypothesized that MPT0B291-inhibited HDACs activity increases acetylation/activation of p53, which in turn results in the induction of cell death, cell cycle arrest as well as a reduction in proliferation.

## Materials and Methods

### Cell culture

Uppsala 87 malignant glioma (U-87MG), a human glioblastoma cell line (ATCC® HTB-14^TM^) was obtained from Invitrogen (Carlsbad, CA, USA) and C6, a rat glioma cell line (BCRC60360) was obtained from the cell bank of the Bioresource Collection and Research Center (Hsinchu, Taiwan). The cell lines were cultured in high-glucose Dulbecco's modified Eagle's medium (DMEM; Gibco, Auckland, NZ) supplemented with 10% heat-inactivated fetal bovine serum (FBS; Biological Industries, Israel) and 1% penicillin-streptomycin (Gibco) 18.

Human astrocytes-hippocampal (HA-h) cell line was used as control (normal) cells for U-87MG and was obtained from Sciencell (Carlsbad, CA, USA) and was cultured in astrocyte medium (Carlsbad) supplemented with 10% heat-inactivated FBS (Biological Industries) and penicillin-streptomycin-glutamine (Gibco, NZ) 19.

A primary culture of rat neuron /glia (N/G) was used as our control (normal cells) for C6 glioma cells. The N/G cultures contain mixed cell populations with astrocytes (47-51%) as the largest population as we previously described 20. Briefly, the brain hemispheres of newborn rats (1-2 day-old) were isolated, washed and homogenized in DMEM (Gibco, NY, USA). Cells were then plated at a density of 5×10^5^ cells/mL, and cultured at 37 °C with 5% CO_2_ and 95% air. Fourteen days following culture initiation the primary neuron/glia cells were used for the experiments described below.

### Plasmid and luciferase gene transduction of U-87MG cells

Luciferase-expressing U-87MG cells are established by transfection of a pGL4.51 [luc2/CMV/Neo] vector (Promega, Madison, WI, USA) into the human glioblastoma cell line U-87MG. The culture medium was changed to the complete medium supplemented with 10% FBS and 1% PS, 17 h after the transfection. Stable cells were selected in the culture medium. The resulting cell line is referred to Luc-U87MG 21.

### Drugs and chemicals

The MPT0B291 (compound 12) and SAHA were synthesized 15 and TMZ was purchased from Formosa Laboratory ltd. (Taoyuan, Taiwan). Stock solutions (10 mM) of MPT0B291, SAHA and TMZ were prepared by dissolving each compound in phosphate-buffered saline (PBS) with 10% dimethyl sulfoxide (DMSO; Bioshop Canada Inc., Ontario, Canada) and were stored at -20 ºC for use in *in vitro* experiments. Stock solutions (15 mg/mL) of MPT0B291, SAHA or TMZ were also prepared by dissolving each compound in 1% carboxymethyl cellulose sodium salt (CMC, Sigma, MO, USA) and 0.5% Tween 80 (Avantor, PA, USA) for oral administration.

### Assessment of cell viability by MTT assay and cell death by lactate dehydrogenase (LDH) activity

All cells were treated with compounds at concentration 1, 3, 10, 30 and 100 µM for 24 h, 48 h and 72 h after growing U-87MG and C6 cells (5000 cell/well) for 18 h and HA-h cells for 24 h and neuron glia mixed primary cells for 14 days, in cell viability (Figure 2A) and lactate dehydrogenase (LDH) activity experiment (Figure 4A). The cultured cells were subjected to the MTT assay. The viability of U-87MG cells and HA-h cultures was measured using the colorimetric 3-(4, 5-dimethylthianol-2-yl)-2,5-diphenyltetrazolium bromide (MTT) reduction assay (Sigma) as previously described. At 24, 48 and 72 h, the medium of the cells was collected for LDH assay 22.

The activity of LDH released from the cells into the medium of the U-87MG, C6, HA-h cells and neuron/glia cultures was measured as previously described. LDH activity was calculated as a slope of the decrease in the OD at 340 nm over 3 min 23. LDH release was expressed as the absolute OD values.

### Assessment cell proliferation by flow cytometry and cell cycle analysis

Cell cycle analysis was performed using a flow cytometer, cell sorter, and cell preparation system (Model no: A00-1-1102; Beckman Coulter, Indianapolis, IN, USA) and the ModFit LT 5.0 software package (Verity Software House, Topsham, ME, USA) was used for analysis. After drug exposure for 24, 48 and 72 h, the cells were collected fixed with 70% ice-cold ethanol and incubated for 30 min on ice. Then, cells were incubated with 10 mg/ml of propidium iodide and 5 mg/ml of ribonuclease-A in PBS for 45 min at 37 °C. ROS and 8-oxo-2'-deoxyguanosine levels were measured as described previously 24. The doublet was excluded manually (Figure S2) and cell cycle gating was performed by auto analysis function of the software.

Flow cytometric determination of apoptosis by Alexa Flour 488 Annexin V/Dead cell apoptosis kit (Thermo Fischer, Oregon, USA) was performed using BD FACS Verse^TM^ Flow Cytometer (Cat. No: 651153). After drug exposure for 24, 48 and 72 h, the cells (1×10^6^ cells/ml) were collected in ice-cold PBS buffer and incubated with Alexa Flour 488 Annexin V and propidium iodide for 15 min and fluorescence emission at 530 nm and 575 nm was measured by flow cytometer cell sorter (Beckman Coulter) as described manufacturer. Data were analyzed and illustrated by BD FACSuite software version 1.0. Double discrimination was shown in supplemental material (Figure S2).

### HDAC activity assay

The enzyme activity of HDAC class I and II was measured using the HDAC-Glo I/II assay kit (Promega, Madison, USA) according to the manufacturer's instruction. Briefly, 1-2×10^4^ cells were suspended and cultured in a white-walled 96-well plate for 4 h, and three-fold serial dilutions (0.001, 0.002, 0.005, 0.014, 0.041, 0.123, 0.370, 1.111, 3.333, 10 µM) of MPT0B291 or SAHA dissolved in assay buffer were added and incubated at 37 °C for 30 min. The HDAC-Glo reagent was then added to each well, and the luminescence signal was measured after incubation at room temperature for 30 min.

HDAC6 enzyme activity was measured using a fluorometric HDAC6 activity assay kit (BioVision, CA, USA), according to manufacturer's instruction. Briefly, 1-2×10^6^ cells were lysed in 100 μL of lysis buffer and centrifuged at 16000 ×*g* for 10 min. Two-fold serial dilution (0.020, 0.039, 0.078, 0.156, 0.312, 0.625, 1.25, 2.5, 5, 10 µM) of MPT0B291, SAHA, tubacin, and lysates were mixed and incubated in white-walled 96-well plates at 37 °C for 10 min. The HDAC6 substrate was then added and the fluorescence signal (excitation/emission = 380/490) was measured after incubation at 37 °C for 30 min 15.

### Orthotopic xenograft and allograft animal models of glioma

The animals were housed in a temperature (21-25 °C) and humidity (45%-50%)-controlled room under a 12-h light/dark cycle and, the animals had *ad libitum* access to pellet chow and water, according to the *International Guidelines for Animal Research*, and the study design was approved by the Institute of Animal Care and Use Committee of Taipei Medical University (LAC20130080). MPT0B291 (M291) was administered by oral gavage.

For xenograft, total 100 male athymic nude mice at 5-6 weeks old (approximately 20 g in body weight) were purchased from BioLasco (Taiwan). Animals were randomized into five groups (Vehicle, MPT0B291, SAHA, TMZ, and MPT0B291+TMZ) and were anesthetized and luciferase-transferred U-87MG cells (Luc-U87MG) were inoculated into brain intracranially according to previously described 18. Briefly, a suspension of 10^6^ Luc-U87MG cells in 2 μl phosphate-buffered saline (PBS)/mice were stereotaxically implanted at the right 2 mm lateral to the bregma and 2 mm below to the surface of the mice skull. Date of dead animals was recorded and Kaplan-Meier survival curve was plotted.

For allografts, total 24 male Sprague-Dawley rats aged 5-6 weeks and weighing approximately 300 g were purchased from BioLasco (Taiwan). Animals were randomized into four groups: (1) Sham surgery + vehicle treatment; (2) Sham surgery + MPT0B291; (3) C6 cells inoculated + vehicle (Veh); and (4) C6 cells inoculated + MPT0B291. The rats were anesthetized and the C6 cells were inoculated intracranially, as previously described 18. Briefly, a suspension of 1×10^7^ C6 cells in 20 μl PBS/rat (20 μl of 5×10^8^ C6 cells/mL suspended cells in PBS) was stereotaxically implanted at the right cortex (2 mm posterior and 3 mm lateral to the bregma) and 3 mm below to the surface of the rat brain. The sham animals received anesthesia, craniotomy and saline injection instead of tumor cell implantation. While recovering anesthesia, the rats were placed in a heated cage to maintain their body temperature. All animals were sacrificed 21 days after tumor cell implantation and ipsilateral (right) and contralateral (left) sides of the brain were collected for further additional experiments.

### Drug administration

MPT0B291, SAHA, and TMZ were dissolved in the solvent to achieve a concentration of 15 mg/mL and were orally administered to the animals in the treatment groups (10 or 25 mg/kg, *p.o.* for MPT0B291 and TMZ, 150 mg/kg, *p.o.* for SAHA) 1 week after tumor implantation. The rational for using two doses was provided by previous publication 15. The animals in the vehicle group received the drug solvent.

### Hematoxylin and eosin staining

Brain tissue samples were excised and fixed in 10% formalin for 24 h and in 75% alcohol for 7 days at 4 °C. The fixed brain samples were embedded in paraffin blocks. Serial sections (3 μm) of the cerebral cortex were stained with hematoxylin and eosin (H&E) for microscopic evaluation. Images were acquired with a visible microscope (Olympus, BX50, Tokyo, Japan) and were analyzed using Spot software (Diagnostic Instruments, Sterling Heights, MI, USA) 25.

### Immunocytochemistry and immunohistochemistry

Immunocytochemistry (ICC) and immunofluorescence (IF) staining for determining the degree of proliferation and apoptosis in the C6 cells was performed as previously described 23. The cultured cells were fixed with 10% formaldehyde for 15 min and incubated with appropriate antibody: rabbit anti-Ki67 (1:200, AnaSpec, San Jose, CA, USA), cleaved caspase-3 (1:200, Cell Signaling, Danvers, MA, USA) and GFAP (1:200, GeneTex). After washing the primary antibody, the cells were incubated with an appropriate biotinylated secondary antibody or appropriate fluorescence secondary antibody and visualized using the avidin-biotin-peroxidase complex method (ABC Elite kit, Vector Laboratories, Burlingame, CA, USA).

Immunohistochemistry (IHC) was performed as previously described 23. The paraffin-embedded tissue sections were dewaxed with xylene and dehydrogenized with alcohol. The citrate buffer (pH 6.0) was used antigen retrieval and tissue sections were incubated with an appropriate primary antibody: rabbit anti-Ki67 (1:200, AnaSpec, San Jose, CA, USA) and cleaved caspase-3 (1:200, Cell Signaling, Danvers, MA, USA). After washing the primary antibody, tissue sections were incubated with an appropriate biotinylated secondary antibody and visualized using the ABC method (Vector Laboratories).

### Western blotting

Cell lysates were prepared by suspending the cells in radioimmunoprecipitation assay (RIPA) buffer (50 mM Tris-HCl at pH 7.4, 1% nonidet P-40, 150 mM NaCl, 1 mM EGTA, 0.025% sodium deoxycholate, 1 mM NaF, 1 mM Na3VO4, and 1 mM PMSF). Equal amounts of protein were electrophoretically separated on 10% sodium dodecyl sulfate-polyacrylamide gels and were transferred to polyvinylidene difluoride membranes (Millipore, CA, USA). The membranes were probed with appropriate antibodies: rabbit polyclonal anti-HDAC6 (1:1000; Abcam), rabbit polyclonal anti-acetylated tubulin (1:1000; Sigma), mouse polyclonal anti-tubulin (1:1000; Abcam), rabbit polyclonal anti-acetylated-histone H3 (1:1000; Millipore), rabbit polyclonal anti-histone H3 (1:1000; Abcam), rabbit polyclonal anti-acetyl-p53 (Lys373, Lys382; Catalog # 06-756) (1:1000; Millipore), rabbit monoclonal anti-phospho-p53 (Ser15; Catalof # 12571) (1:1000; Cell Signaling, MA, USA), rabbit polyclonal anti-PUMA (1:1000; Abcam), rabbit polyclonal anti-Bax (1:1000; Abcam), rabbit polyclonal anti-Apaf1 (1:1000; Bio Vision), mouse polyclonal anti-p53 (1:1000; Abcam), mouse monoclonal anti-β-actin (1:2000; GeneTex, Hsinchu, Taiwan), and were quantified using colorimetric substrates.

### Quantitative RT-PCR

Total RNA was extracted from C6 cells by using the TRIzol reagent (Invitrogen, Life Technologies, Carlsbad CA, USA), according to manufacturer's instruction. The concentration and purity of the extracted RNA were determined by measuring ultraviolet absorbance at 260 and 280 nm. For reverse transcription (RT), RNA samples (3 μg) was used in a total reaction volume of 20 μl with ReverTra Ace set (Purigo, Taipei, Taiwan) and complementary DNA (cDNA) synthesis, as previously described 26.

For detection of mRNA expression, the templates were amplified using a Rotor-Gene SYBR Green PCR Kit (Qiagen) on a Rotor-Gene Q 2plex HRM Platform (Qiagen). The primers used for the qRT-PCR assay were as follows: Caspase-3, 5′-AATTCAAGGGACGGGTCATG-3′ (forward) and 5′-GCTTGTGCGCGTACAGTTTC-3′ (reverse); PUMA, 5′-CATGGGACTCCTCCCCTTAC-3′ (forward) and 5′-CACCTAGTTGGGCTCCATTT-3′ (reverse); Bax, 5′-GTGAGCGGCTGCTTGTCT-3′ (forward) and 5′-GTGGGGGTCCCGAAGTAG-3′ (reverse); Apaf1, 5′-GGCCATCTGAGACATTCCA-3′ (forward) and 5′-CATGAGCTGAAGGGCCATA-3′ (reverse); p21, 5′-ACATCTCAGGGCCGAAAAC-3′ (forward) 5′-GCGCTTGGAGTGATAGAAAT-3′ (reverse); Cdk2 5′-CCTGCACCAGGACCTCAAG-3′ (forward) 5′-CGGTGAGAATGGCAGAATG-3′ (reverse); Cdk4 5′-ACAGCTACCAGATGGCCCT-3′ (forward) 5′-CAGCCTCAGAGTTCCCACA-3′ (reverse); Cdk6 5′-AGTGTTGGCTGCATCTTTG-3′ (forward) 5′-CCTGTCTGGGAAGAGCAAC-3′ (reverse); β-actin, 5′-GACCCAGATCATGTTTGAGACCTTC-3′ (forward) and 5′-GGTGACCGTAACACTACCTGAG-3′.

Following reaction conditions were maintained for 40 cycles (5 min at 95 °C for initial denaturation, 5 s at 95 °C and 10 s at 60 °C for denaturation). The relative expression of selected mRNA transcripts was calculated using the comparative cycle threshold (∆∆Ct) method and normalized to the expression of β-actin or GAPDH 27.

### *In silico* analysis

REpository for Molecular BRAin Neoplasia DaTa (REMBRANDT) online platform database (http://betastasis.com/glioma/rembrandt/) was used to determine gene expression based on HDAC transcript levels using the online Project Betastasis representation tool.

### Statistical analysis

Data of MTT, LDH assay and flow cytometry among compound or concentration groups, and data of Western blot, qRT-PCR between the groups (Veh, M291) were evaluated using Student *t*-test and one-way analysis of variance (ANOVA). Protein and mRNA expression between the vehicle and treatment groups were statistically analyzed using the Student *t*-test. All statistical analysis were conducted and bar graph displays were created using Sigma Stat and Plot version 2.0 (Jandel Scientific), and the *p-value* cut-off was set at 0.05. Data are presented as mean ± standard error of mean (SEM).

## Results

### mRNA levels of HDACs were increased in human GBM tissue

**Figure 1:**
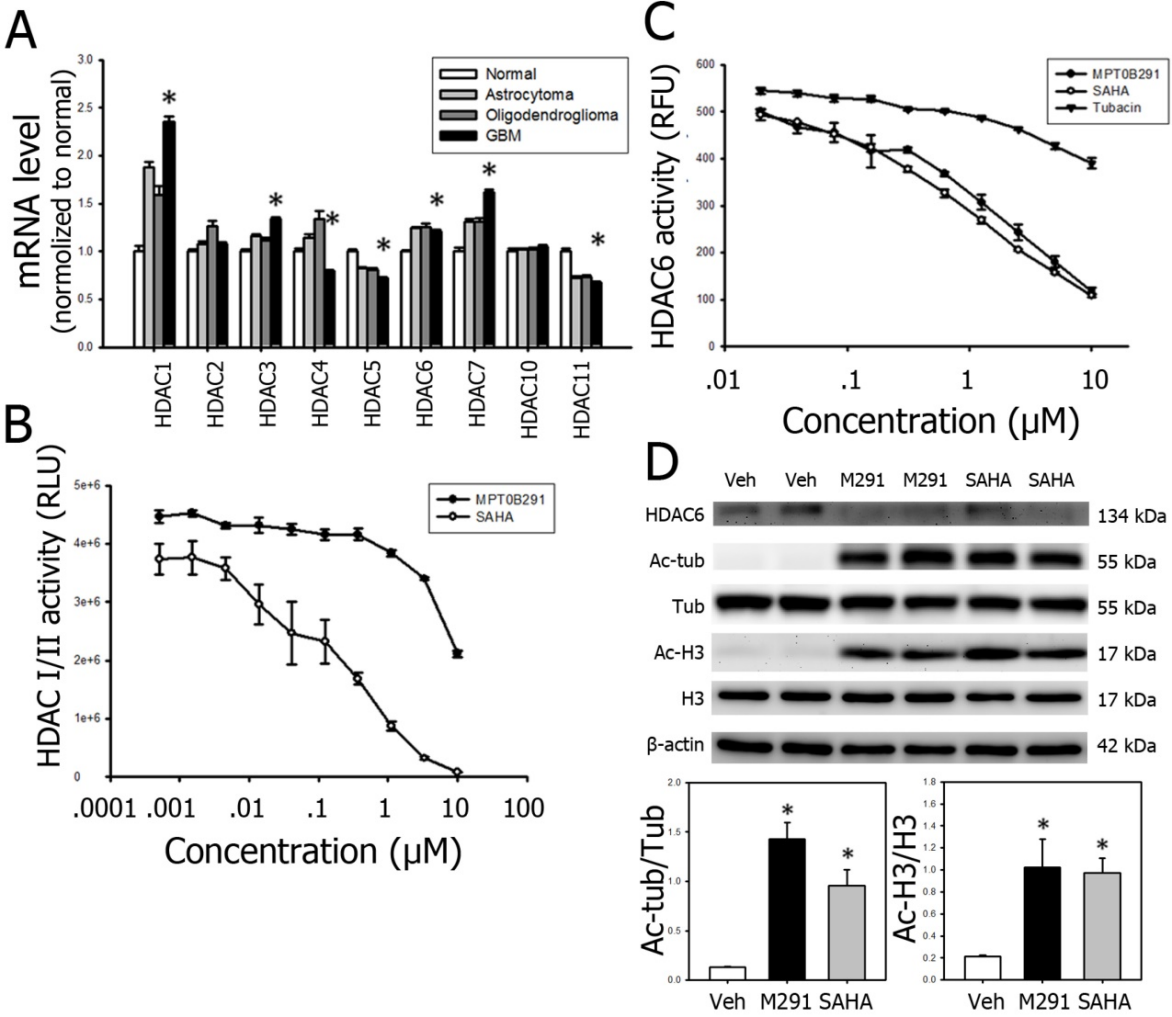
MPT0B291 has pan-HDAC inhibitory effect in long term**.** (**A**) The relative mRNA expression of HDACs was obtained from REMBRANDT and is presented as mean ± SEM. *p<0.05 versus the normal cells. (**B**) The enzyme activity of HDACs was measured on C6 cells treated with MPT0291 or SAHA at threefold serial diluted concentration using an HDAC-Glo I/II assay kit at 30 min (suggested by manufacturer). Data is presented as mean ± SEM (n=3 in each group). (**C**) The enzyme activity of HDAC6 was measured on C6 cells treated with MPT0291 or SAHA at twofold serial diluted concentration using the fluorometric assay kit at 10 min (suggested by manufacturer). Data is presented as mean ± SEM (n=3 in each group). (**D**) Representative images of Western blot analysis for HDAC6, acetyl-tubulin and acetyl histone H3 in the C6 cells treated with vehicle (Veh), MPT0B291 and SAHA at concentration of 30 µM for 24 h. The relative protein levels of acetyl-tubulin and acetyl histone H3 in vehicle (Veh)-, MPT0B291- and SAHA-treated the C6 cells are presented as mean ± SEM. (n=4 in each group) *p<0.05 versus the vehicle-treated group.

We first used an online platform data base (http://betastasis.com/glioma/rembrandt/) and compared the mRNA expression levels of some HDACs in adjacent normal tissue, and tissue from astrocytoma, oligodendroglioma and GBM. Among 11 HDACs compared, the mRNA expression levels of HDAC 1, 3, 6, and 7 were significantly increased by 88.3%, 16.4%, 24.0%, and 30.8%, respectively; the mRNA expression levels of HDAC 4, 5 and 11 were significantly decreased by 20.7%, 28.2% and 32.8% in GBM tissue, respectively (Figure 1A).

### MPT0B291 has relatively selective inhibitory effect on HDAC6 in 10 min and pan-HDAC inhibitory effect in later time point

To examine whether MPT0B291 inhibits HDACs on glioma cells, we conducted HDAC activity assays (Figure 1B-C) and confirmed by the expression of acetyl-tubulin (a consequence of cytoplasmic inhibition) and acetyl-histone H3 (a consequence of nuclear inhibition) in C6 glioma cells (Figure 1D). The results of HDAC-Glo I/II activity assay showed that MPT0B291 inhibited activities of HDAC class I (HDAC 1, 2, 3, 8) and class II (HDAC 4, 5, 6, 7, 9, 10) in a concentration-dependent manner; however, the inhibition by MPT0B291 was not as potent as SAHA inhibition (Figure 1B). To further clarify whether MPT0B291 selectively inhibits HDAC6, we performed an HDAC6 activity assay (fluorometric) in the C6 cells. MPT0B291 as well as SAHA inhibited HDAC6 in a concentration-dependent manner much potent than tubacin 28, a well-known HDAC6 inhibitor (Figure 1C). It suggests that SAHA is more potent than MPT0B291 in inhibition of class I and class II HDACs (HDAC 1-10) while MPT0B291 is as potent as SAHA in HDAC6 inhibition. Protein level of acetyl-Histone H3 and acetyl-tubulin were both increased after MPT0B291 treatment with significantly increased level of acetyl-tubulin (7.1 folds) and acetyl-histone H3 (2.0 folds) (Figure 1D).

MPT0B291 is relatively selective in HDAC6 inhibition in glioma cells within 10 min (suggested by manufacturer of HDAC6 activity assay kit) (Figure 1C). However, MPT0B291 has pan-HDAC inhibitory effect evidenced in later time point by Western blot detection (Figure 1D).

### MPT0B291 significantly reduced cell viability in human and rat glioma cells, but not in normal human and rat astrocytes

**Figure 2:**
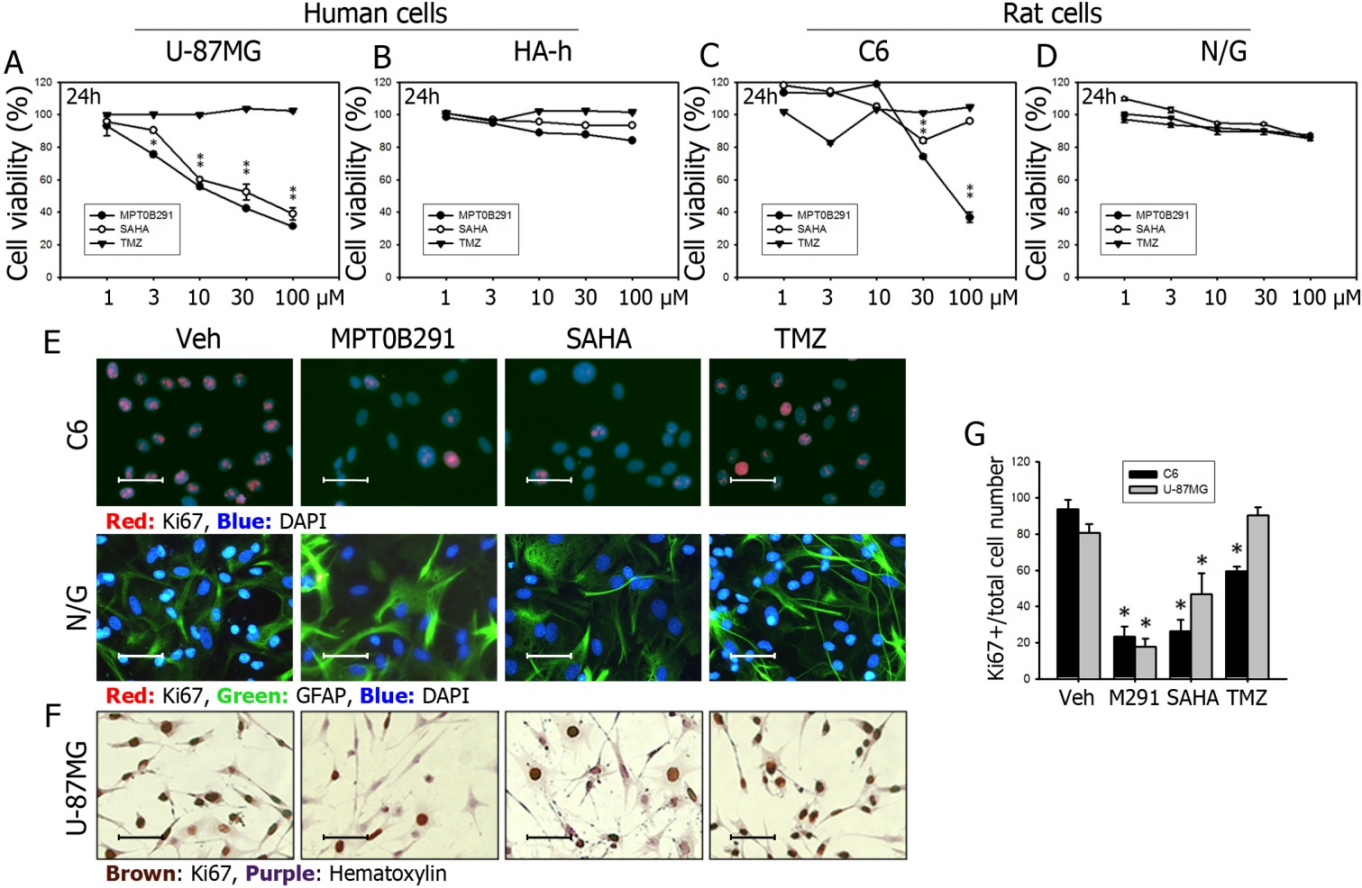
MPT0B291 reduces the proliferation of U-87MG and C6 cells but not in the human astrocytes. The U-87MG human glioma cells versus human hippocampal astrocytes (HA-h) and C6 rat glioma cells versus neuron/glia mixed primary culture were used, and cell viability was measured using the MTT assay at 24 in U-87MG (**A**), HA-h (**B**), C6 cells (**C**) and primary culture (N/G) (**D**). (**E**) Representative images of immunofluorescence (IF) staining for Ki67 on the C6 and neuron/glia primary culture cells treated with vehicle (Veh), MPT0B291 (30 µM), SAHA (30 µM) and TMZ (30 µM) for 24 h. The Ki67 is shown in red and GFAP is shown in green. **(F)** Representative images of immunocytochemistry (ICC) for Ki67 on the U-87MG cells. The Ki67 is shown in brown (Calibration = 50 µm). **(G)** The percentage of Ki67-positive cells is normalized with total C6 cell number and presented as mean ± SEM (n=4 in each group) *p<0.05 versus the vehicle-treated group.

We found that MPT0B291 was more effective than SAHA in reducing the viability of human glioblastoma U-87MG cells (Figure 2A), but not human normal astrocytes (Figure 2B) at 24 h as indicated by MTT assay. MPT0B291 (30 μM) and SAHA (30 μM) significantly reduced the viability of U-87MG cells by 42.2% and 52.5%, respectively. The same concentration of TMZ did not exhibit a significant effect on the cell viability (Figure 2A). Similar results were observed for rat glioma C6 cells (Figure 2C), but not primary culture of rat neuron-astrocyte (Figure 2D). MPT0B291 (30 μM) and SAHA (30 μM) significantly reduced the viability of C6 cells by 74.1% and 84.1%, respectively (Figure 2C). However, MPT0B291 did not reduce the viability of human astrocytes (HA-h) and rat primary culture as markedly as SAHA did. The 50% inhibitory concentration (IC50) of MPT0B291 for the viability of U-87MG cells were 65.3, 9.1, and 2.2 μM at 24 h, 48 h, and 72 h, respectively, and the IC50 for viability of C6 cells was 16.1, 2.1, and 1.9 μM, respectively (Table 1).

### MPT0B291 significantly inhibited proliferation in glioma cells

Glioblastoma is astrocytic tumor and GFAP is a cell-type specific marker for astrocytes. While we used HA-h (human astrycytes-hippocampal) cells as control (normal) cells for U-87MG, we used primary culture of neuron/glia (N/G) as our control (normal cells) for C6 glioma cells. We did observe that GFAP was all positive in U-87 and C6 glioblastoma cells and Ki67 (a marker of proliferation) was all negative in N/G mixed cells. A supporting evidence for the all GFAP-positive glioblastoma can be seen in Supplemental Figure 1 (Figure S1) showing the all GFAP-positive staining in U-87MG xenografted mouse brain sections with or without the treatment of MPT0B291. We then double-stained Ki67 and GFAP in C6 and N/G cells and found that all three compounds decreased Ki67-possitive proliferating cells in C6 (Figure 2E) as well as U-87MG glioblastoma cells (Figure 2F). The quantitative results in C6 and U-87MG cells also confirmed that MPT0B291, SAHA or TMZ all significantly inhibited the proliferation of glioblastoma cells (Figure 2G).

**Figure 3:**
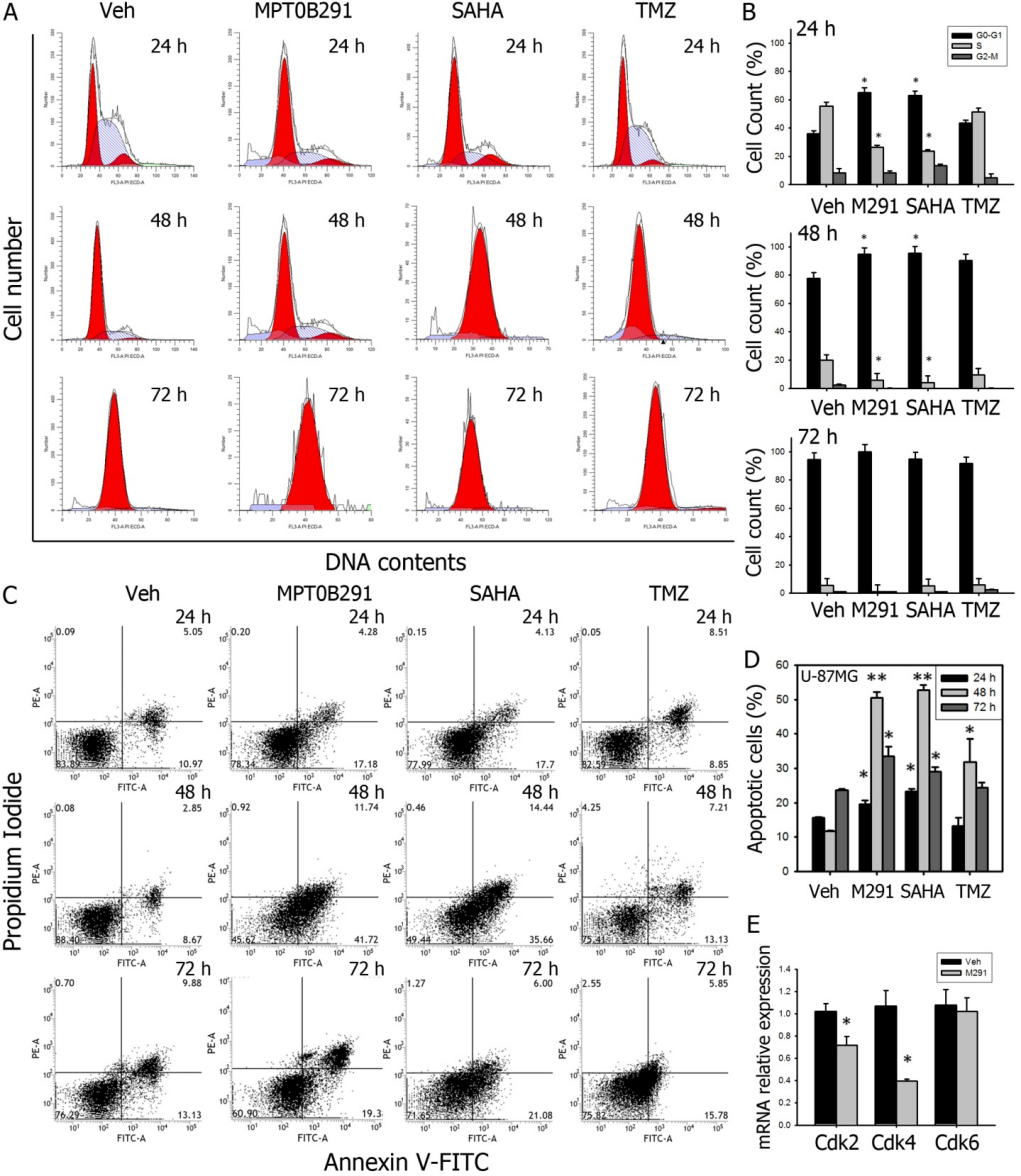
MPT0B291 induces G1 cell cycle arrest and apoptosis in U-87MG cells **(A)** Representative histograms showing flow cytometry analysis of cell cycle analysis on U-87MG cells treated with vehicle, MPT0B291 (30 µM), SAHA (30 µM) and TMZ (30 µM) for 24 h, 48 h and 72 h. **(B)** The percentage of the U-87MG cells in different cell cycle stages is presented as mean ± SEM (n=3 in each group). **(C)** Representative histograms showing flow cytometry analysis of Annexin V/PI stained U-87MG cells treated with vehicle, MPT0B291 (30 µM), SAHA and TMZ for 24 h, 48 h and 72 h. **(D)** Percentages of apoptosis cell population are presented as mean ± SEM (n=3 in each group). **(E)** The relative mRNA expression levels of Cdk2, Cdk4 and Cdk6 in vehicle- and MPT0B291-treated C6 cells are presented as mean ± SEM (n=3 in each group) *p<0.05 versus vehicle-treated group.

To further confirm the effect of MPT0B291 on proliferation of glioma cells, we performed cell cycle analysis by using flow cytometry (Figure 3A). Data showed that MPT0B291 and SAHA significantly increased proportion of U-87MG cells in G1 phase by 80.1% and 74.4% at 24 h comparing to vehicle-treated cells; and significantly decreased proportion of U-87MG cells in S phase by 2.1 and 2.3 times at 24 h, respectively (Figure 3B) These results indicated that MPT0B291 or SAHA induced the G1 arrest of the U-87MG cells. MPT0B291 also down-regulated mRNA levels of G1/S regulators: Cdk2 and Cdk4, significantly (Figure 3E). Taken together, these results indicate that MPT0B291 inhibited cell proliferation by inducing G1 cell cycle arrest and down-regulating G1/S regulators Cdk2 and Cdk4 in glioma cells.

### MPT0B291 significantly promoted cell death in both human and rat glioma cells, but not in normal human and rat astrocytes

**Figure 4:**
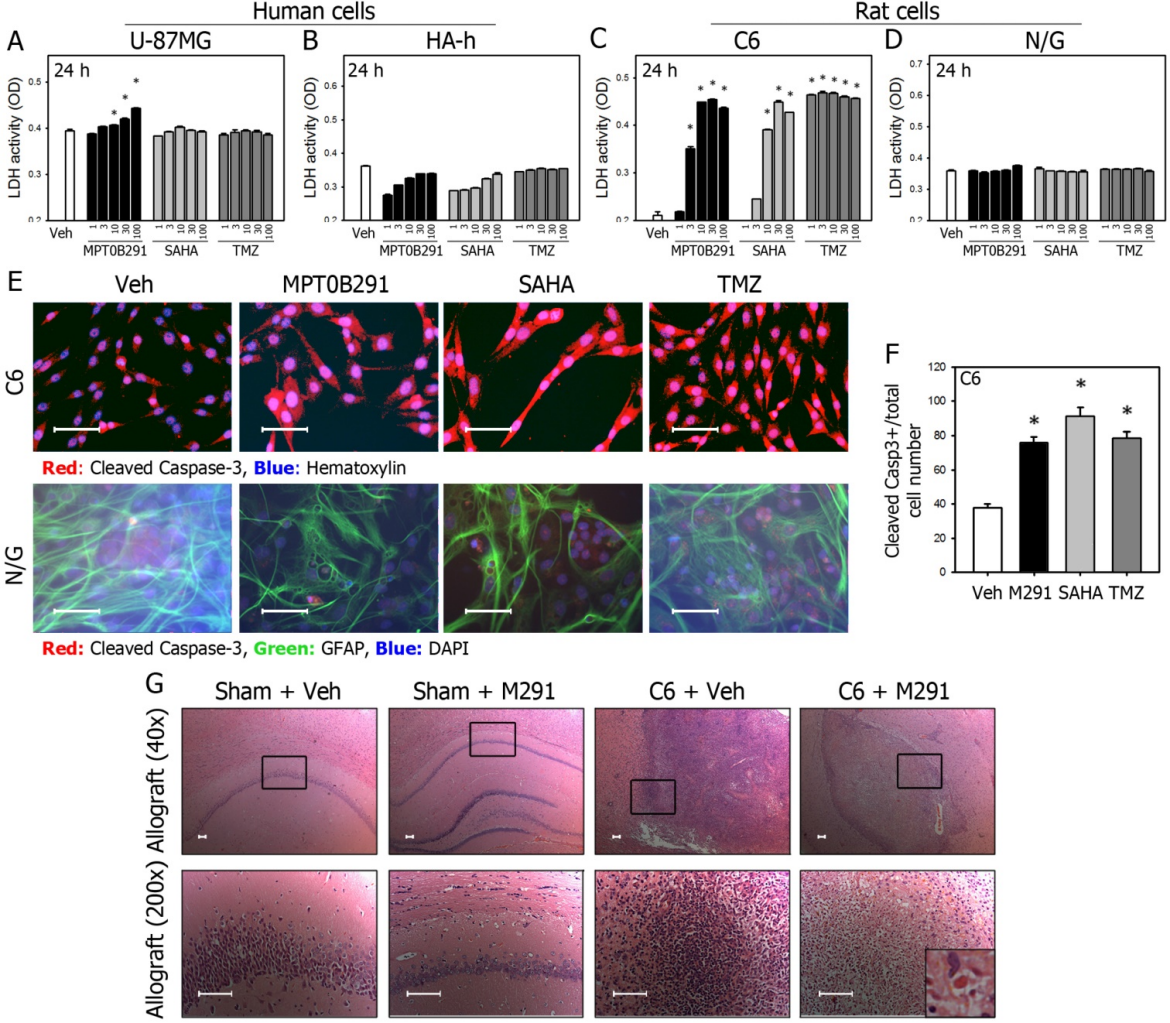
MPT0B291 induces cell death and apoptosis in glioma cells *in vitro* and *in vivo***.** The LDH activity in the medium of U-87MG **(A)**, HA-h **(B)**, C6 **(C)**, and neuron/glia mixed primary culture (N/G) **(D)** treated with various concentrations of MPT0B291, SAHA and TMZ was measured and data are presented as mean ± SEM. (n=3 in each group) *p<0.05 versus vehicle-treated group. **(E)** Representative images of IF for cleaved caspase-3 in the C6 cells and neuron/glia primary culture treated with vehicle (Veh), MPT0B291 (30μM), SAHA (30μM) and TMZ (30 µM) for 24 h. (Calibration = 50 µm). **(F)** The percentage of cleaved caspase-3 positive cells is normalized with total C6 cell number and presented as mean ± SEM (n=4 in each group) *p<0.05 versus the vehicle-treated group. **(G)** Representative images of HE-stained brain sections from sham and allografted animals with or without MPT0B291 treatment. Arrow in the inset indicated apoptotic bodies, which is characteristics of apoptotic cells (Calibration = 50 µm).

The LDH release assay is a standard method for measuring cell death and has been used extensively in the HDAC inhibitor literature 29. MPT0B291 increased lactate dehydrogenase (LDH) activity in the U-87MG (Figure 4A) and C6 cells (Figure 4C), but not in human astrocyte (Figure 4B) and neuron/glia mixed primary culture (Figure 4D). Importantly, LDH activity was increased more in MPT0B291 treated cells than those in SAHA treated U-87MG cells. MPT0B291 (30 μM) significantly increased cell death (measured by LDH activity) of U-87MG cells to 24.1% at 24 h, respectively. MPT0B291 (30 μM) and SAHA (30 μM) significantly increased LDH activity by the C6 cells to 115.2% and 112.6% at 24 h, respectively (Figure 4A, C).

To confirm the anti-tumor activity of MPT0B291, apoptosis of U-87MG cells was analyzed by flow cytometric Annexin V/PI assay (Figure 3C). MPT0B291 significantly increased the number of apoptotic cells at all-time points by 1.3 times (24 h), 4.3 times (48 h), 1.4 times (72 h) and SAHA significantly increased this number by 1.5, 4.5 and 1.2 times (Figure 3D).

To examine whether MPT0B291 promotes apoptosis in the glioma, we performed IF (Figure 4E). More cleaved Caspase-3-positive cells were observed in glioma cells than those in normal astrocytes (GFAP-positive cells). The quantitative results showed that MPT0B291 and SAHA increased percentage of cleaved Caspase-3 positive C6 cells 2.0 and 2.4 time, respectively (Figure 4F). Taken together, the results indicate that MPT0B291 promoted apoptosis in the U-87MG and C6 cells but not in the human and rat astrocytes.

### MPT0B291 reduced tumor growth and induced cell death in C6-allografted rats

To evaluate the anti-tumor effects of MPT0B291 *in vivo*, we established allograft as well as xenograft animal models. Following the oral administration of MPT0B291, C6-implanted allograft rats were sacrificed and the brains were fixed. In HE-stained brain sections, the tumor cells in C6 + Veh group could be easily identified not only by increased size of nuclei but also by higher nucleus-to-cytoplasm (N/C) ratio showing more blue (hematoxylin) than red (eosin). Apoptotic cells as identified by apoptotic bodies showing dense cytoplasm containing fragments of disintegrated nucleus and surrounded by light, 'hollow' space were observed in C6 + M291 animals but not C6 + Veh animals implicating that MPT0B291 induced apoptosis in tumor cells *in vivo* (Figure 4G).

### MPT0B291 reduced tumor growth and induced cell death in U-87MG xenografted mice

**Figure 5:**
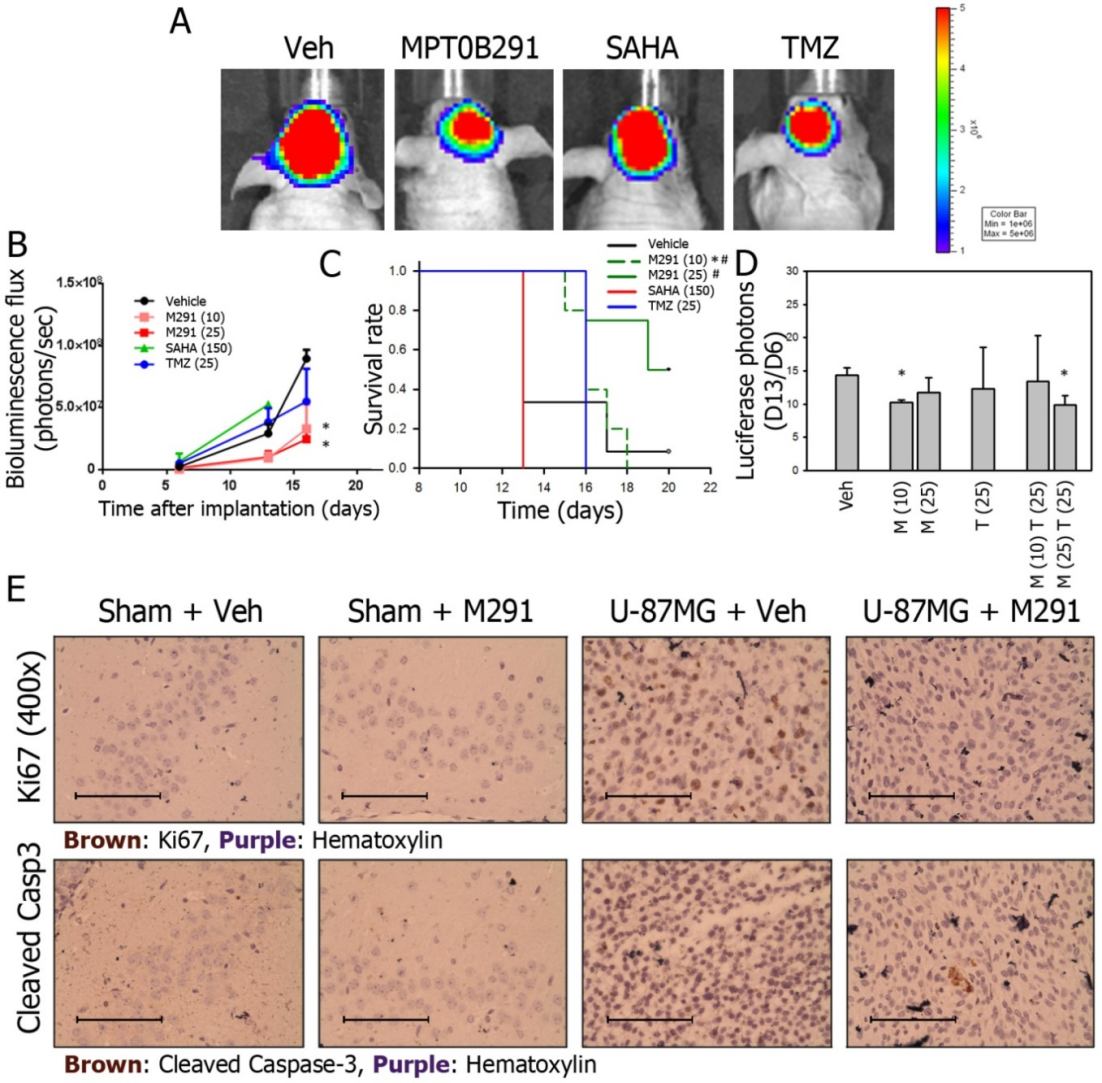
MPT0B291 reduces glioma growth in the xenograft animal model. (**A**) Representative IVIS200 images of xenografts treated with vehicle (Veh), MPT0B291 (25 mg/kg, *p.o.*), SAHA (150 mg/kg, *p.o.*) and TMZ (25 mg/kg, *p.o.*). (**B**) The bioluminescence flux of xenografts treated with vehicle, MPT0B291 (10 or 25 mg/kg, *p.o.*), SAHA (150 mg/kg, *p.o.*) and TMZ (25 mg/kg, *p.o.*) at d6, d13 and d16 is presented as mean ± SEM (n=4 in each group). (**C**) Kaplan-Meier survival curve of xenografts treated with vehicle, MPT0B291 (10 or 25 mg/kg, *p.o.*), SAHA (150 mg/kg, *p.o.*) and TMZ (25 mg/kg, *p.o.*), *p<0.005 versus Vehicle; p<0.005 versus SAHA (150). (**D**) The normalized bioluminescence flux (day13/day6) of xenograft treated with MPT0B291 (10 or 25 mg/kg, *p.o.*), TMZ (25 mg/kg, *p.o.*) and MPT0B291 plus TMZ is presented as as mean ± SEM (n=4 in each group). **(E)** Representative images of IHC for Ki67 (brown) and cleaved caspase-3 (brown) in brain section of sham mice and U-87MG-xenografts treated with or without MPT0B291. (Calibration = 50 µm).

IVIS200 imaging system is often used to demonstrate that quantitative bioluminescence (BLU) flux is proportional to the number of live tumor cells expressing luciferase. BLU flux correlates directly with tumor size. In our study, BLU flux (tumor size) was reduced the most in MPT0B291 groups (10 and 25 mg/kg, *p.o.*) compared to the vehicle-treated group at 16th days of tumor transplantation (Figure 5A, 5B). SAHA failed to reduce the tumor size at a dose up to 150 mg/kg, (*p.o.*) (Figure 5B) and SAHA-treated animals only survived 13 days (Figure 5C). In animals treated with MPT0B291 (10, 25 mg/kg, *p.o.*), the tumor sizes were significantly smaller (Figure 5B) and the survival time was longer than 20 days (Figure 5C).

To further evaluate whether MPT0B291 can be used as an adjuvant to chemotherapy in glioma cells, we treated xenograft animals with combination of MPT0B291 (10 or 25 mg/kg, *p.o.*) and TMZ (25 mg/kg, *p.o.*) based the results shown in Figure 5B. The results showed that treatment of MPT0B291 (10 or 25 mg/kg, *p.o.*) improved the efficacy of TMZ (25 mg/kg, *p.o.*) but MPT0B291 was not synergistic with TMZ in reducing tumor size (Figure 5D).

We also assessed the effects of MPT0B291 (25 mg/kg, *p.o.*) on tumor cell proliferation (by Ki67 staining) and apoptosis (by cleaved caspase-3 staining) in sham (without tumor) and U-87MG xenografted mice, More Ki67-possitive cells were observed in brain sections from U-87MG xenografted mice than those from sham mice. MPT0B291 (25 mg/kg, *p.o.*) reduced proliferating cells in U-87MG xenografted mice (Figure 5E, top). However, apoptotic cells (cleaved caspase-3-positive) were only observed in brain sections from xenografted mice with MPT0B291 treatment and were hardly seen vehicle-treated xenograft mice (Figure 5E, bottom).

### MPT0B291 increased acetylation and phosphorylation of p53 *in vitro* and *in vivo*

**Figure 6:**
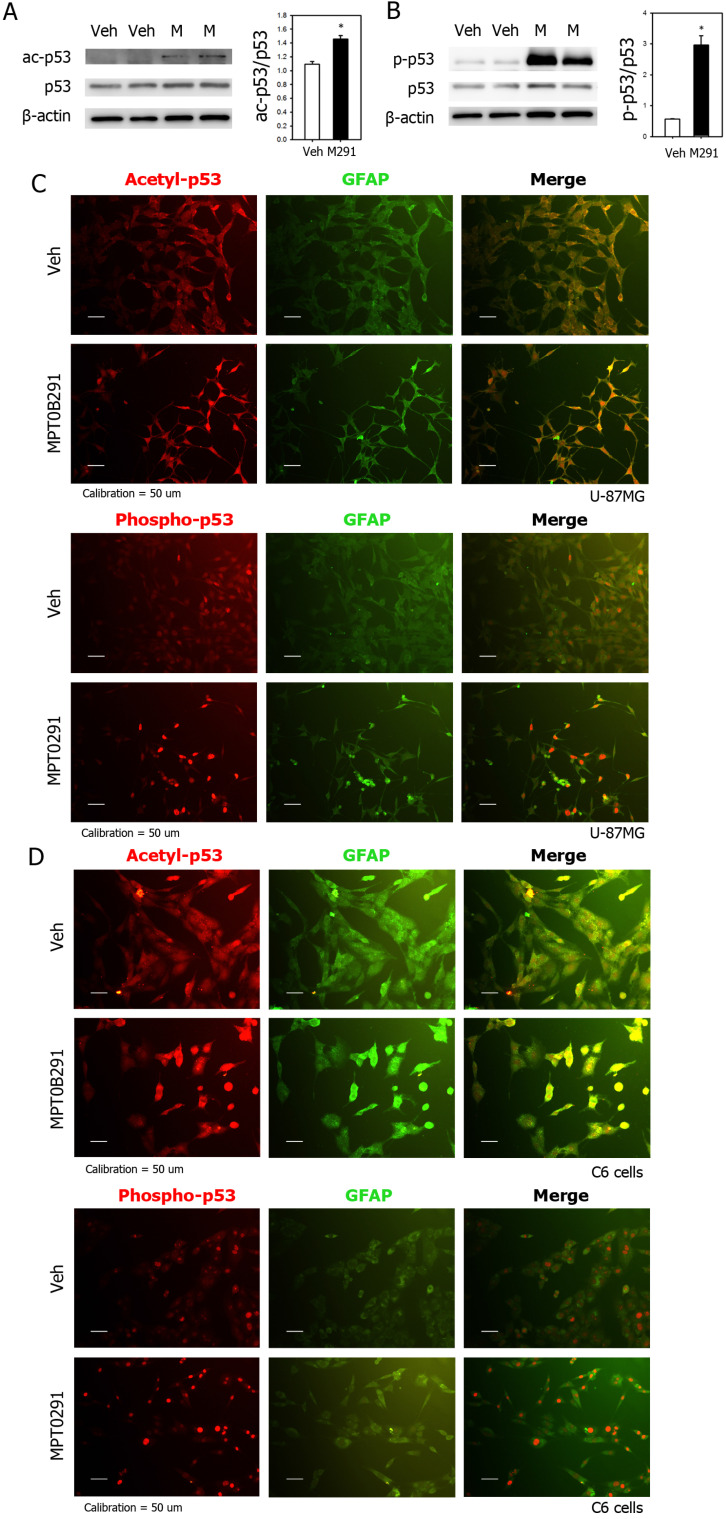
MPT0B291 increases acetylation and phosphorylation of p53 in U-87MG and C6 cells. (**A**) Representative images of Western blot analysis for acetyl-p53. (**B**) Representative images of Western blot analysis for phospho-p53. The relative protein levels are presented as mean ± SEM (n=4 in each group). Representative images of IF for acetylated and phosphorylated p53 in the U-87MG (**C**) and C6 (**D**) cells treated with vehicle (Veh) and MPT0B291 (30 µM). The acetylated and phosphorylated p53 are shown in red, GFAP in green and Dapi in blue. (Calibration = 50 µm).

MPT0B291 also significantly increased acetylation of p53 by 1.3 times shown by expression levels of acetyl-p53 in Western blots (Figure 6A). Particularly, it dramatically increased phosphorylation of p53 by 5.3 times (Figure 6B). To show localization of acetylated (top) and phosphorylated p53 (bottom), we performed IF staining on U-87MG (Figure 6C), C6 cells (Figure 6D) *in vitro* and brain sections of xenografted mice (Figure S1). The result shown that MPT0B291 increased acetylation and phosphorylation of p53 in both U-87MG (Figure 6C) and C6 cells (Figure 6D). The acetylated p53 is mostly localized both nucleous and cytoplasm and phosphorylated p53 is localized within nucleus *in vitro* (Figure 6C-D) as well as in xenografted Luc-U87MG cells *in vivo* (Figure 6C-D) and *in vivo* (Figure S1).

### MPT0B291 increased expression of genes related to apoptosis and cell cycle arrest

**Figure 7:**
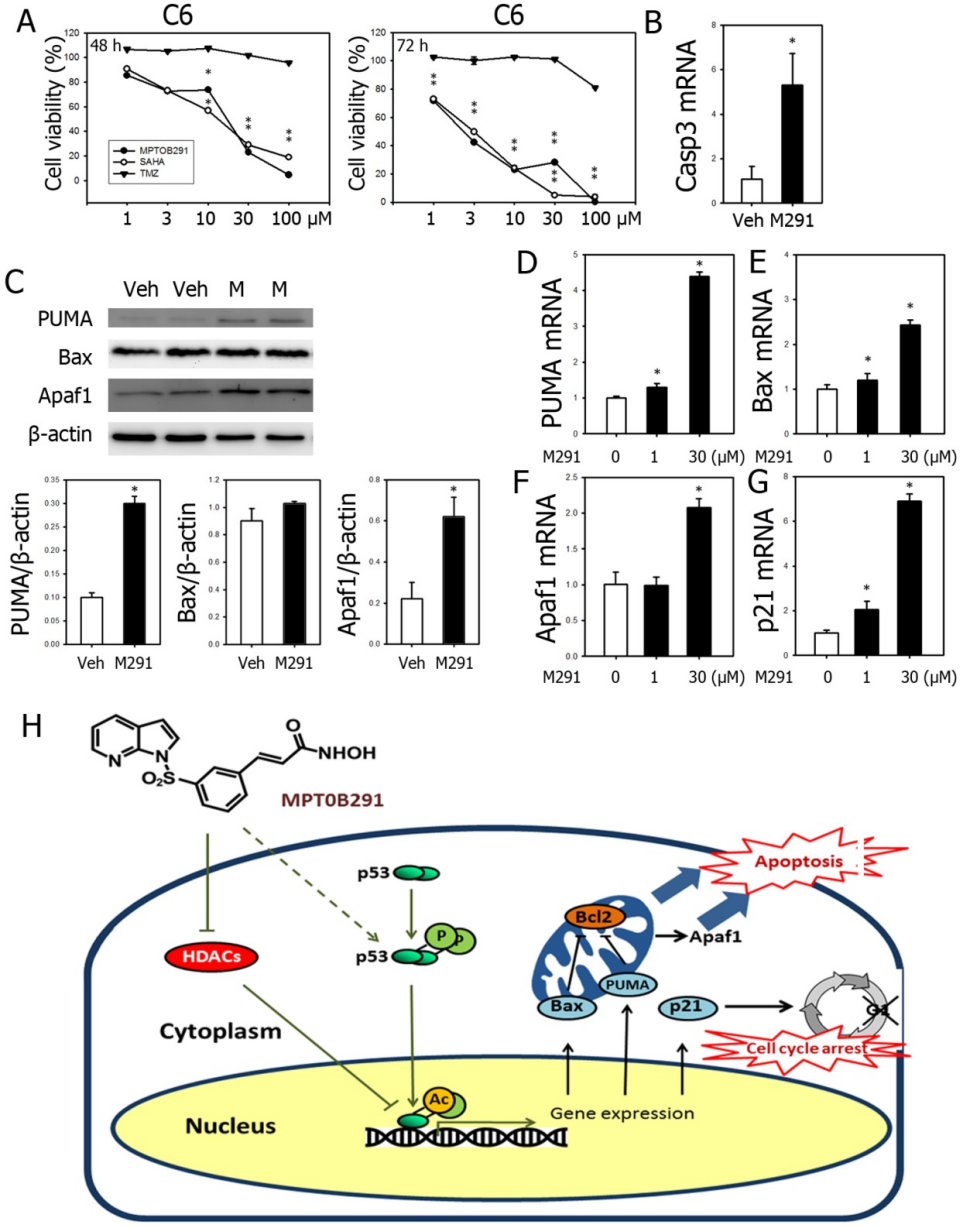
Reduced viability of glioblastoma cells was simultaneously observed with increased mRNA expression of PUMA, Bax, Apaf1 and p21 in MPT0B291-treated glioma cells. (**A**) Anti-tumor activities were evaluated using MTT assay comparing with SAHA and TMZ at 24, 48 and 72 h (See also Figure 2A). (**B**). The relative mRNA expression levels of caspase-3 in C6 cells with or without treatment are presented as mean ± SEM (n=3 in each group) (**C**) Representative images of Western blot analysis for PUMA, Bax and Apaf1. The relative protein levels are presented as mean ± SEM (n=4 in each group). The relative mRNA expression levels of PUMA (**D**), Bax (**E**), Apaf1 (**F**) and p21 (**G**) in vehicle- and MPT0B291-treated groups are presented as mean ± SEM (n=3 in each group) *p<0.05 versus the vehicle-treated group. **(H)** Schematic diagram of mechanistic studies. MPT0B291 inhibited HDAC activity leading to partially increased acetylation/activation of p53 as well as phosphorylation of p53, which in turn results in the induction of cell death, cell cycle arrest as well as a reduction in proliferation.

MPT0B291 inhibited cell viability in concentration-dependent and time-dependent manner (Figure 2A and 7A), as well as induced expression of Caspase-3 (Figure 7B). To investigate the molecular mechanisms underlying the effects of MPT0B291, we quantified expression of apoptosis-related genes, PUMA, Bax, Apaf1, and cell cycle arrest-related gene, p21 in the C6 cells. MPT0B291 significantly increased protein level of PUMA by 3 times and Apaf1 by 2.8 times (Figure 7C). MPT0B291 (30 µM) significantly increased the relative mRNA expression of PUMA by 4.38 times, Bax by 2.42 times, Apaf1 by 2.06 times and p21 by 6.86 times (Figure 7D-G).

## Discussion

Previous studies have shown that HDAC inhibitors induce cell death 17, 28, 30, and cell cycle arrest 17, 28, 31-33 and suppress cell proliferation 17, 28, 30-37, and angiogenesis 38. HDAC6-selective inhibitors, tubastatin A, CAY10603, and ACY1215 inhibited cell proliferation and induced apoptosis of U-87MG, U251, and A172 cells by inhibiting HDAC6 34 and HDAC inhibitors sensitized glioblastoma cells to TMZ and radiotherapy 34, 39-43. Our study showed that a novel HDAC inhibitor MPT0B291 also inhibited cell proliferation and induced cell death in the U-87MG and C6 cells.

The activity of various HDACs is required for the survival of glioma cells 44. Using the online platform database (http://betastasis.com/glioma/rembrandt/) we found expression of HDAC1, HDAC3, HDAC6, and HDA7 up-regulated in astrocytoma, oligodendroglioma, and GBM (Figure 1A). Using another database (https://www.oncomine.org/) we already showed HDAC1/3/6/9 were overexpressed in brain malignancy 45. Regarding HDAC class III enzymes (Sirt 1-7), it has been demonstrated that Sirt1 protein level is decreased in glioblatoma comparing to normal astrocyte 46 and Sirt7 in both mRNA and protein level is increased in glioma 47. Previous studies showed that U-87MG and C6 cells express HDAC 1, 2 and 6 48-52.

HDAC6 is an important target for neuroprotection and neuroregeneration in traumatic brain injury, stroke and neurodegenerative disorders 11-14 and HDAC inhibitors reduce the cytotoxic effects of anti-tumor agents in normal cells 53, 54. Previous study suggested that over expression of HDAC6 is related to drug resistance of GBM 45. Because of stronger enzymatic effect (two catalytic domains) and neuro-protective feature, we targeted HDAC6 and used a novel HDAC inhibitor, MPT0B291. In this study, we showed that the novel HDAC inhibitor, MPT0B291 was less toxic against primary astrocytes and neuron/glia mixed primary culture. While we did not expect normal cells to proliferate as quickly as U-87MG glioma cells, we expected these cells to die by apoptosis and necrosis. No neurological toxicities were detected in the *in vivo* study.

HDAC inhibitors cause the deacetylation histone as well as non-histone proteins 6, 55. SAHA induces the p38-mediated activation of p53 in glioblastoma stem-like cells (GSCs) 56. A study verified that HDAC1 blockade prevented the development of radio-resistance in the U-87MG cells by reactivating the p53 protein 57. The pan-HDAC inhibitor, DWP0016, activates p53 transcription and acetylation and to inhibited U251 cell growth 58. Consistent with these results, p53 acetylation/activation by MPT0B291 were associated with induced cell death and suppressed cell proliferation in the U-87MG and C6 cells.

Trichostatin A also increases the phosphorylation of p53 and the expression of Bax 56. In medulloblastoma, p53-dependent Bax activation and apoptosis were increased after treatment with HDAC inhibitor and chemotherapy 57. In our study, MPT0B291 increased the expression levels of PUMA, Bax, Apaf1, and p21 in the C6 cells.

Abrogation of G1 arrest and re-entered cell cycle was observed in the p53-depleted U-87MG cells. The cyclin-dependent kinase inhibitor p21 was the major player in trichostatin A-induced cell cycle arrest. This effect was accompanied by the significant up-regulation of p53 and its transcriptional target p21 20. In our study, acetylation of p53 and simultaneous up-regulation of p21 and induction of G1 arrest was observed in the U-87MG cell treated with MPT0B291 similar to the effect of trichostatin A. Since previous publication indicated elevation of Caspase-3 and reduction of p21 protein level 20, we did not perform Western blot for Caspase-3 and p21. Pai JT et al. 2015 hypothesized that acetylated-Hsp90 disrupts cyclin-dependent kinases and following degradation 60. In our study, mRNA level of Cdk2, Cdk4 and Cdk6 down-regulated in MPT0B291-treated glioma cells indicating that HDAC6 inhibitor transcriptionally regulates those cyclin-dependent kinases.

We found that MPT0B291 is highly specific HDAC6 (Figure 1B-C); However, continuous inhibition of HDAC6 by MPT0B291 affected HDAC I/II (Figure 1D). These results may suggest that HDAC6 inhibition may affect HDACI/II activity, but these effects require additional time to manifest. Future studies will require determining the potential link between HDAC6 and HDACI/II activities. Based on HDAC inhibition and suppresses of tumor cell viability, we used 2 different concentrations of MPT0B291 (1 μM or 30 μM) and in molecular study. Results showed that mRNA level of PUMA, Bax, Apaf1 and p21 were increased by MPT0B291. Further study used p53 knock-down model is required.

The p53 is deacetylated at C-terminal lysines K320, K373 and K382 by HDAC1 while at lysine K382 by SIRT1. Other HDACs (2, 3 and 6) may also down-regulate p53 acetylation and function although more evidence is needed to establish those interactions and their biological significance 16. Interestingly, acetylation of p53 at K382 and K381 prevents p53 from inducing apoptosis in mouse primary cortical neuron. In our study, we indicated that a novel HDAC inhibitor MPT0B291 causes increased acetylation at lysines K373 and K382 and it was toxic to human and rat glioma cells, but not normal astrocytes. It was consistent with recently published work showing that HDAC6 deacetylates p53 at lysines 381/382 and differentially coordinates p53-induced apoptosis 61.

We also showed that MPT0B291 inhibited tumor growth in allograft and xenograft animal models. In our pilot study used xenografted mice, we originally used same doses for each compounds. Our preliminary IVIS200 bioluminescence photon data showed that dose of SAHA was always not enough to affect the tumor cell growth. So, we increased dose of SAHA in real experiments up to 150 mg/kg (*p.o.*). Not only SAHA at such a higher dose failed to reduce the tumor size, but also it was toxic so that SAHA-treated animals only survived 13 days. Our data clearly indicated that MPT0B291 at smaller dose (10 mg/kg, *p.o.*) than SAHA and TMZ has better effects on animal survival and on inhibiting glioblastoma growth *in vivo*.

In summary, enhanced tumor cell death and reduced tumor cell proliferation as well as increased acetylation of p53, and increased mRNA expression of PUMA, Bax and p21 were simultaneously observed with HDAC inhibition in MPT0B291-treated glioma cells (Figure 7H). Although U-87MG (human) and C6 (rat) glioma cell lines used in this study are both with wild type p53, existing literature indicates that the mutation status of p53 gene has not been correlated with survival in GBM patients 62-64. Since high mutation frequency of p53 was also found in GBM.

## Conclusion

In conclusion, our preclinical study showed that the novel HDAC inhibitor, MPT0B291 inhibited cell growth *in vivo*. Mechanistical studies showed that MPT0B291 promoted cell death, cell cycle arrest and reduced proliferation, as well as increased phosphorylation and acetylation/activation of p53 in glioma cell lines. Therefore, HDAC inhibitor MPT0B291 represents a potential new therapeutic strategy for gliomas.

## Supplementary Material

Supplementary figures.

## Figures and Tables

**Table 1:** Inhibitory concentration 50 (IC50, µM) of MPT0B291, SAHA and TMZ on cell viability of U-87MG and C6 cells

Exposed time	24 h	48 h	72 h
**U-87MG cells**			
MPT0B291	16.05	2.15	1.85
SAHA	37.47	3.46	4.19
TMZ	*NCR*	*NCR*	*NCR*
**C6 cells**			
MPT0B291	65.40	9.08	2.25
SAHA	*NCR*	13.07	2.97
TMZ	*NCR*	*NCR*	*NCR*

NCR, not in concentration range.
