# Supplementary figures and images for: Histone deacetylase inhibitor MPT0B291 suppresses Glioma Growth in vitro and in vivo partially through acetylation of p53
Source: Int J Biol Sci. 2020 Oct 19;16(16):3184–99. doi: 10.7150/ijbs.45505 (PMC7645997; doi:10.7150/ijbs.45505)

Figure S1

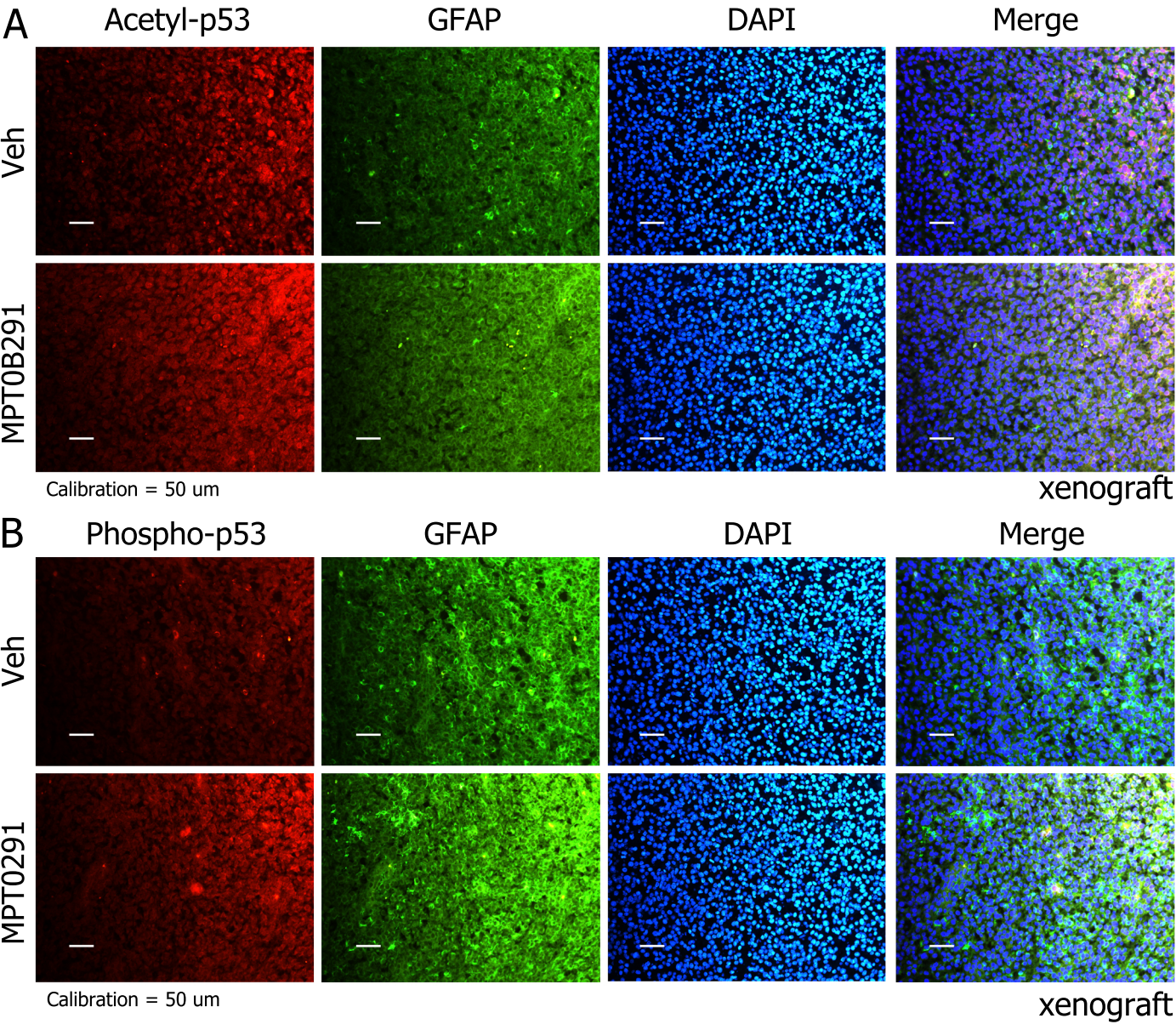

Figure S2

A

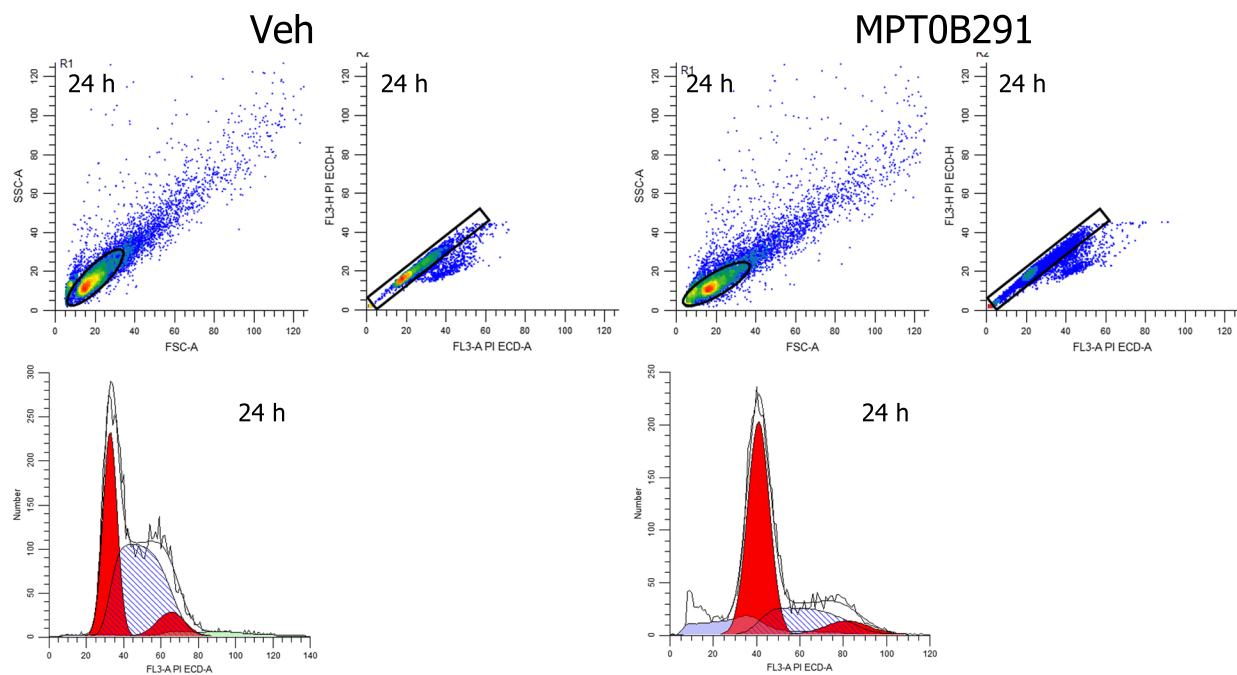

B

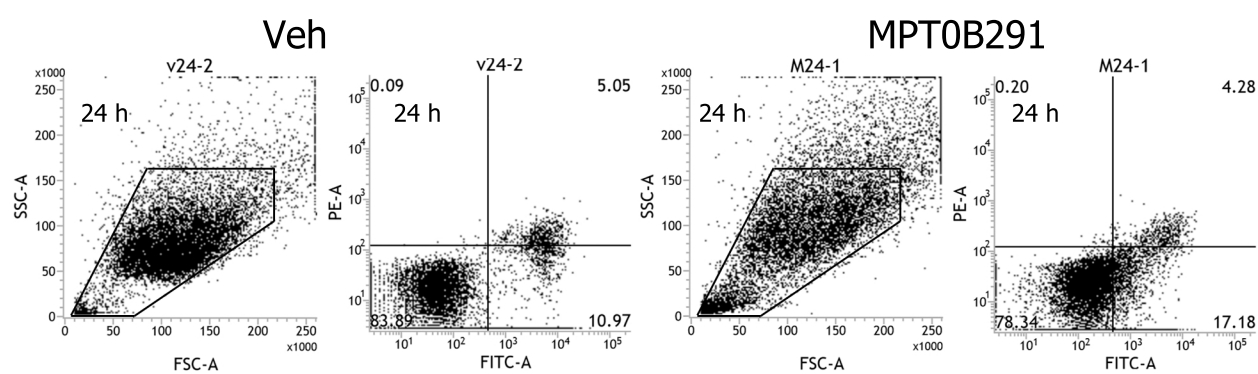

Supplement: Supplementary file 1 — Supplementary figures. [file ijbsv16p3184s1.pdf]
